# Strategies of *Helicobacter pylori* in evading host innate and adaptive immunity: insights and prospects for therapeutic targeting

**DOI:** 10.3389/fcimb.2024.1342913

**Published:** 2024-02-26

**Authors:** Jiawei Fan, Jianshu Zhu, Hong Xu

**Affiliations:** ^1^ Department of Gastroenterology, The First Hospital of Jilin University, Changchun, China; ^2^ Department of Spine Surgery, The First Hospital of Jilin University, Changchun, China

**Keywords:** *Helicobacter pylori*, immune evasion, adaptive immunity, microRNA, vaccination

## Abstract

*Helicobacter pylori* (*H. pylori*) is the predominant pathogen causing chronic gastric mucosal infections globally. During the period from 2011 to 2022, the global prevalence of *H. pylori* infection was estimated at 43.1%, while in China, it was slightly higher at approximately 44.2%. Persistent colonization by *H. pylori* can lead to gastritis, peptic ulcers, and malignancies such as mucosa-associated lymphoid tissue (MALT) lymphomas and gastric adenocarcinomas. Despite eliciting robust immune responses from the host, *H. pylori* thrives in the gastric mucosa by modulating host immunity, particularly by altering the functions of innate and adaptive immune cells, and dampening inflammatory responses adverse to its survival, posing challenges to clinical management. The interaction between *H. pylori* and host immune defenses is intricate, involving evasion of host recognition by modifying surface molecules, manipulating macrophage functionality, and modulating T cell responses to evade immune surveillance. This review analyzes the immunopathogenic and immune evasion mechanisms of *H. pylori*, underscoring the importance of identifying new therapeutic targets and developing effective treatment strategies, and discusses how the development of vaccines against *H. pylori* offers new hope for eradicating such infections.

## 1 Introduction

*H. pylori*, a Gram-negative, microaerophilic, helical bacterium, is considered one of the most successful human pathogens (Camilo et al., 2017). Recent data indicates a global *H. pylori* prevalence of approximately 43.1% between 2011 and 2022 (Li et al., 2023), with a slightly higher infection rate of 44.2% in China (Ren et al., 2022), surpassing the global average. Infection typically occurs in early childhood, with a global pediatric infection rate of 32.3% (Kalach et al., 2017). Despite eliciting strong innate and adaptive immune responses in hosts, *H. pylori* persists, inducing chronic inflammation that can lead to gastritis, peptic ulcers, and malignancies such as mucosa-associated lymphoid tissue (MALT) lymphomas and gastric adenocarcinomas (Baj et al., 2021; Yang and Hu, 2022). Chronic gastritis, often asymptomatic, is predominantly caused by *H. pylori* infection, which is also a principal cause of gastric and duodenal ulcers. Notably, *H. pylori* infection is a major independent risk factor for gastric cancer, with approximately 1-2% of those infected progressing to cancer. Gastric cancer is currently the sixth most common cancer and the fourth leading cause of cancer-related deaths globally, posing a significant health threat (Salvatori et al., 2023).

*H. pylori* maintains a stable presence in specific ecological niches within the stomach, a capability intricately linked to its array of virulence factors. Urease activity disrupts the gastric acid barrier, facilitating survival in acidic conditions (Stingl et al., 2002). *H. pylori*’s motility, mediated by its flagella (Gu, 2017), further promotes colonization on the gastric mucosal surface. Outer membrane proteins like BabA, SabA, AlpA/B, and OipA, along with lipopolysaccharides (LPS), mediate adherence to gastric epithelial cells (Tshibangu-Kabamba and Yamaoka, 2021; Valkonen et al., 1994), contributing to colonization and pathogenesis. The vacuolating cytotoxin A (VacA) disrupts cellular integrity by inducing vacuole formation, while the high-temperature requirement A protease (HtrA) cleaves key intercellular adhesion proteins like E-cadherin, occluding and claudin-8, impairing epithelial barrier functions (Ansari and Yamaoka, 2020; Albrecht et al., 2018). Notably, Cag pathogenicity island (PAI)-encoded virulence factor CagA manipulates signaling pathways such as ERK/MAPK, NF-KB, leading to pathologic alterations in cell morphology, adhesion, polarity, proliferation, and motility (Wang et al., 2023). Additionally, the presence of CagA-positive *H. pylori* infection has been closely associated with the severity of gastric cancer and adverse clinical outcomes in affected patients (Parsonnet et al., 1997). Host factors including environmental influences and genetic polymorphisms may also augment *H. pylori*’s colonization efficiency and susceptibility to related diseases. The traditional quadruple therapy, including proton pump inhibitors, amoxicillin, clarithromycin, and bismuth agents, has long been recommended as the first-line treatment for global *H. pylori* eradication (Lu et al., 2023). However, rising antibiotic resistance has reduced eradication rates, with recent rates falling below 80% in many countries (Tshibangu-Kabamba and Yamaoka, 2021), necessitating the development of novel therapeutic strategies.

**Figure 1:**
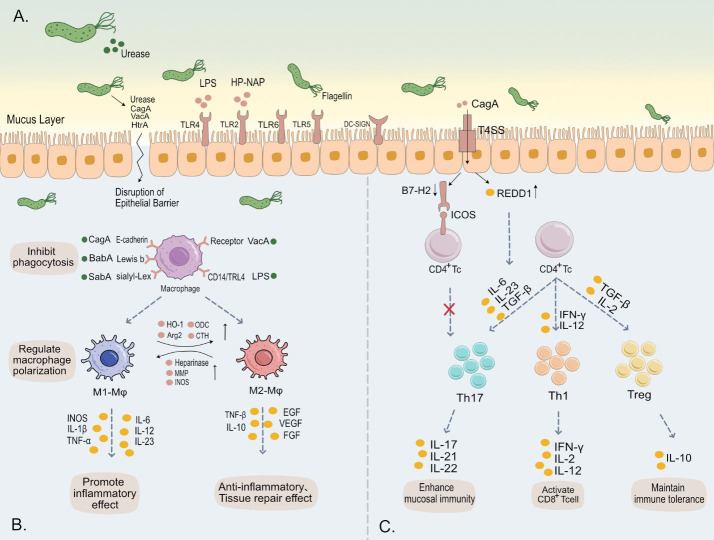
**(A)** *H*. *pylori* employs urease to neutralize gastric acidity, while its flagella and helical shape facilitate traversal through the gastric mucus layer. Subsequently, a multitude of virulence factors of *H*. *pylori*, such as LPS, flagella, and CagA, bind to various receptors on the gastric epithelial surface, triggering intracellular immune responses. Additionally, these virulence factors are capable of evading immune system activation and disrupting the tight junctions of gastric epithelium. This leads to damage of the gastric mucosa, contributing to the pathogenesis of *H*. *pylori*-associated gastric disorders. **(B)** Upon binding of *H*. *pylori* outer membrane proteins and virulence factors to surface receptors on macrophages, there is an inhibition of phagocytosis in these immune cells. This interaction further modulates the polarization and immune metabolism of macrophages, thereby significantly impacting the host’s innate immune response. **(C)** The release of various cytokines induces differentiation in CD4+ T cells into subsets such as Th1, Th17, and Treg, maintaining immune balance. *H*. *pylori* specifically influences this differentiation towards Tregs, fostering cell-mediated immune tolerance. This adaptation by *H*. *pylori* is crucial for its persistent colonization and chronic infection in the host.

*H. pylori* infection presents a significant clinical challenge, as this bacterium can establish persistent colonization within the host despite a robust immune response, leading to a reduced eradication success rate in patients (Karkhah et al., 2019). Existing studies have revealed strategies employed by *H. pylori* to evade the host’s immune system through specific molecular mechanisms, but a comprehensive understanding of these mechanisms is still limited, especially in terms of how they are precisely regulated to adapt to different host environments. Furthermore, the mechanisms by which host genetic and physiological factors influence the immune evasion response of *H. pylori* remain unclear and warrant further investigation. Multiple potential therapeutic targets have been identified in the mechanisms of *H. pylori* ‘s evasion of immune responses. Additionally, vaccines targeting *H. pylori* have shown promising results in preclinical trials, with these vaccines preventing infection by activating and modulating immune cell phenotypes, demonstrating significant potential for future development (Friedrich and Gerhard, 2023). This review will explore the strategies employed by *H. pylori* to manipulate the host’s immune system for chronic colonization (**Figure 1**), and emphasize the importance of new therapeutic strategies and *H. pylori* vaccines targeting these mechanisms.

## 2 Crossing the gastric acid mucosal layer to achieve colonization

The gastric cavity possesses an extremely acidic pH (typically between 1 to 2), which is not an ideal environment for bacterial colonization (Ramsay and Carr, 2011). Additionally, the gastric mucosal layer serves as a physical barrier that not only prevents bacterial penetration into deeper tissues but also entraps antimicrobial compounds within the host, thereby enhancing resistance to bacterial infections (Shimizu et al., 1996). Therefore, *H. pylori* ‘s initial colonization in the stomach relies on its urease activity and motility to buffer its cytoplasm and escape from the gastric lumen into the mucous layer, reaching a more favorable environment with a higher pH (**Table 1**).

**Table 1:** Mechanisms of immune evasion by virulence factors of *H. pylori* and resulting effects.

Virulence factor	Mechanism of escape immunity	Effect	Reference
Urease	1. Decompose urea into ammonia and carbon dioxide 2.Regulates MLC phosphorylation and occludin endocytosis in tight junctions 3.Stimulates Macrophages to induce nitric oxide synthase expression	1. Neutralizes stomach acid 2.Disrupts gastric epithelial tight junctions 3.Chemotaxis of Neutrophils and Monocytes	(Stingl et al., 2002; Wroblewski et al., 2009; Gobert et al., 2002)
Bacterial shape	Helical bacterial shape	Promotes *H. pylori* to cross the acidic mucus layer of the stomach	(Constantino et al., 2016)
Flagella	1.Motility 2.Flagellin recognizes TLR5 and drives NF-κB Activation 3.R89T, L93K, and E114 mutations impair the interaction between flagellin’s TLR5 activation hot spot residues and TLR5’s LRR9 loop cavity	1.Promotes *H. pylori* to cross the acidic mucus layer of the stomach 2.Activates the innate immune response 3.Escapes immune recognition	(Gu, 2017; Zou et al., 2021; Hayashi et al., 2001; Gewirtz et al., 2001; Kim et al., 2018)
SabA	Binds with sialyl-Lex antigen	Adhesion to gastric epithelial mucosa	(Doohan et al., 2021)
BabA	Binds with Lewis b antigen	Adhesion to gastric epithelial mucosa	(Doohan et al., 2021)
LPS	1. Lipid A reacts with protons in stomach acid 2.Core region binds to th ECM layer’s adhesion proteins, LeX polysaccharide structures in O-antigen interact with galectin-3 3. Recognizes TLR2 and TLR4 Receptors 4. Dephosphorylation of lipid A at the 1’ and 4’ phosphate positions, with longer acyl chains of 16 and 18 carbons O-antigen chain expresses Lewis x and y antigens for molecular mimicry 5.Binds to macrophage surface glycoproteins such as CD14 and TLR4, triggering NF-κB-mediated inflammatory responses 6.stimulates iNOS expression, promoting the conversion of L-arginine to NO, leading to mitochondrial dysfunction	1. Neutralize stomach acid 2.Adhesion to gastric epithelial mucosa 3.Activates the innate immune response 4. Escapes immune recognition 5. Disrupts macrophage phagocytosis 6.Impedes the transformation of M1 into M2 macrophages	(Calam et al., 1997; Fowler et al., 2006; Cullen et al., 2011; Moran, 2008; Wang et al., 2000; Ciesielska et al., 2021; Cherdantseva et al., 2014)
HtrA	Cleaves key cell adhesion proteins such as E-cadherin, occludin, and claudin-8	Disrupts gastric epithelial tight junctions	(Albrecht et al., 2018)
Hp-NAP	Binding to TLR2 induces neutrophils and monocytes to produce IL-8 and ROS	Activates the innate immune response	(Wen et al., 2021)
VacA	1. Vacuolates gastric epithelial cell 2.Increases intracellular Ca^2+ concentration, triggering unfolded protein response (UPR) and endoplasmic reticulum (ER) dysfunction 3.Phosphorylates T-cell surface β2 integrins via PKCη or PKCζ, inducing actin rearrangement for VacA endocytosis and T-cell G1/S cell cycle arrest 4.Induces anti-inflammatory cytokines IL-10, TGF-β gene expression, and TGF-β signaling; activates Wnt/β-catenin pathway and upregulates CCL28	1. Causes cellular vacuolation 2. Disrupts macrophage phagocytosis 3. Inhibits T-cell proliferation 4. Induces Treg cell differentiation	(Akazawa et al., 2013; Sewald et al., 2011; Ji et al., 2020)
CagA	1. Secretes chemokines IL-8 and urease 2. Increases intracellular Ca^2+ concentration 3. Activates p38 MAPK and NRF-2 signaling pathways, inducing increased HO-1 expression 4. Reduces B7-H2 expression through p70 S6 kinase phosphorylation 5. Enhances NF-κB binding to the REDD1 promoter via the MAPKp38 pathway 6. Activates the Wnt/β-catenin pathway and upregulates CCL28 7. Induces downregulation of the let-7 family miRNAs expression	1. Promotes directional movement of *H. pylori* 2.Disrupting macrophage phagocytosis 3. Induces transition from M1 to M2 and Mreg phenotypes 4.Reduces CD4+ effector T-cell activity and increases Treg 5. Induces Th17 cell polarization, exacerbating inflammation 6. Promotes Treg cell differentiation 7.Decreases inflammatory responses	(Ferreira et al., 2016; Wong et al., 2017; Kang et al., 2021; Lina et al., 2013; Yan et al., 2021; Hayashi et al., 2013)
γ-glutamyltransferase	1.Mediates extracellular glutathione cleavage and ROS generation, causing lymphocyte cell cycle arrest. Inhibits cMyc and IRF4 expression, disrupting T-cell proliferation. Interferes with Ras-dependent signaling, leading to G1 cell cycle arrest 2. Inhibits DC maturation and induces T-cell FoxP3/CD25 expression	1. Inhibits T-cell proliferation 2.Induces Treg cell differentiation	(Schmees et al., 2007; Wüstner et al., 2015; Shibayama et al., 2003; Oertli et al., 2013)

The urease produced by *H. pylori* hydrolyzes urea present in the stomach into ammonia and carbon dioxide. The ammonia neutralizes gastric acid, forming ammonium ions, thereby providing a more neutral microenvironment conducive to bacterial survival (Stingl et al., 2002). This helps mitigate the direct bactericidal effect of gastric acid. Furthermore, increased functional urease activity (urease B) and the production of ammonia disrupt the cellular cytoskeleton-mediated tight junctions by regulating the phosphorylation of myosin light chain (MLC) and endocytosis of the tight junction protein occluding, leading to enlarged intercellular spaces (Wroblewski et al., 2009). This allows harmful substances in the gastric fluid to penetrate further into the gastric wall tissue. Gastric mucins have physiologically relevant sol and gel properties. In the acidic environment of the stomach, mucins typically form a gel-like state that effectively captures bacteria. However, in the presence of urease activity, the pH increases, subsequently decreasing the viscosity of the mucins and enabling bacteria to move within the mucous layer (Celli et al., 2009; Celli et al., 2005). Although the primary function of urease in *H. pylori* is to alter pH by producing ammonia, studies have revealed the presence of urease in the lamina propria of individuals infected with *H. pylori*. The interaction between Urease B and CD74 activates NF-κB, leading to increased production of interleukin-8 (IL-8), a potent chemotactic factor for monocytes and neutrophils (Beswick et al., 2006). *H. pylori* urease and functionally active recombinant urease also stimulate the expression of inducible nitric oxide synthase in macrophages (Gobert et al., 2002), which may lead to the production of substances causing nitrosative damage.

Motility is crucial for *H. pylori* infection (O'Toole et al., 2000). Similarly, mutants lacking directed motility exhibit weakened virulence. *H. pylori* exhibits chemotaxis, mediated by a complex chemosensory system comprising multiple receptors like TlpA, TlpB, TlpC, and TlpD, detecting various chemical signals. These signals, relayed through coupling proteins to CheA kinase, alter CheY activity. Active CheY then guides the bacterium’s movement via its flagellar motor, allowing *H. pylori* to adeptly navigate and localize within specific gastric mucosal and glandular niches. This precise localization enables robust colonization during the acute phase of infection (Johnson and Ottemann, 2018; Williams et al., 2007). Mutations in chemotactic receptors TlpA or TlpB of *H. pylori* can lead to reduced or lost chemotaxis (Williams et al., 2007). In addition, mutations that lack the Cag pathogenicity island (PAI) were found in *in vitro* experiments to impair the ability of *H. pylori* to trigger gastric epithelial cells to secrete pro-inflammatory cytokines and chemokines, such as IL-8 and urease (Ferreira et al., 2016). This reduces the number of bacteria approaching gastric epithelial cells, thereby alleviating the severity of gastritis. In such cases, bacteria fail to trigger a strong immune response. The helical shape of *H. pylori* allows it to navigate the gastric mucus layer and facilitates penetration through epithelial cells (Constantino et al., 2016). Its spiral shape is influenced by various proteins, including Csd1/2, Csd5, Csd7, CcmA, and MurF, which directly or indirectly affect the structure and composition of peptidoglycan (PG). Particularly, the rod-shaped protein domain of CcmA and its N-terminal region are crucial for the helical shape, enabling polymerization and interaction with Csd5 and Csd7, playing a key role in maintaining cellular shape stability and regulating cell wall architecture (Sichel et al., 2022). This complex helical structure has been considered by some authors to be a virulence factor. *In vitro* experiments have shown that helical cells exhibit a 15% increase in propulsive force compared to straight mutants. Although the helical cell shape has little effect on swimming speed, the loss of helical shape increases the proportion of bacteria completely immobilized in mucus by 30-40%, potentially affecting the turnover rate of bacterial clearance from the mucus layer (Constantino et al., 2016; Martínez et al., 2016). Moreover, the helical shape increases the proportion of motile bacteria. Beyond cell shape, the number of flagella significantly contributes to the motility speed of *H. pylori*. The flagellar structure of *H. pylori* contributes to effective colonization on the gastric mucosa through mechanisms such as motility, adhesion capability, and penetration through the mucous layer (Gu, 2017).

Lipopolysaccharide (LPS) is a key component on the surface of *H. pylori*, facilitating the bacterium’s invasiveness, interaction with the host, and evasion of gastric mucosal defense mechanisms. The lipid A region of LPS can react with protons in gastric acid, reducing the bactericidal effect of the acid (Calam et al., 1997), thus favoring the survival of *H. pylori* in the gastric environment. Specifically, the core region of LPS binds to host extracellular matrix (ECM) layer adhesins (Valkonen et al., 1994), and the LeX polysaccharide structures in the O antigen are bound by host β-galactoside-binding lectin (galectin-3) (Fowler et al., 2006), which have been proven to participate in the colonization process, laying the groundwork for chronic infection. Additionally, the PAP2 protein HP0851 (HupA), a key enzyme in LPS biosynthesis, is involved in LPS modifications that increase the positive charge of lipid A, thereby enhancing resistance to cationic antimicrobial peptides (CAMP) (Gasiorowski et al., 2019), helping the bacteria evade the host’s innate immune response.

## 3 Evasion of host innate immunity

### 3.1 Evading receptor recognition

The immune response initiated by *H. pylori* begins with the recognition of bacterial pathogen-associated molecular patterns (PAMPs) by pattern recognition receptors (PRRs) expressed on innate immune cells or gastric epithelial cells. These PRRs include toll-like receptors (TLRs), NOD-like receptors (NLRs), C-type lectin receptors (CLRs), and retinoic acid-inducible gene (RIG)-I-like receptors (RLRs), all of which are involved in the identification of *H. pylori* and the activation of innate immunity (Cheok et al., 2022).

Lipopolysaccharide (LPS) is among the most potent activators of the innate immune system, and variations in the bacterial lipid A structure may represent a successful strategy to evade innate immune recognition and impede the TLR4 signaling pathway (Matsuura, 2013). *In vitro* studies suggest that lipid A in *H. pylori* LPS is less likely to be recognized by human TLRs, and chemical modifications that remove negative charges from the 1’ and 4’ phosphate groups of lipid A significantly reduce the potency of LPS. Substituting the phosphate groups with other negatively charged groups, such as phosphoethanol, has minimal impact (Ulmer et al., 1992). This indicates that dephosphorylation increases the chances of evading TLR recognition, but it is not the sole factor. Notably, LPS prepared from a double LpxE/F mutant synthesizing both diphosphorylated and hexa-acylated lipid A still displayed attenuated hTLR4-MD2 activity (Cullen et al., 2011), possibly due to the longer acyl chains (16 and 18 carbons) in the *H. pylori* lipid A. Furthermore, some *in vitro* studies have shown low reactivity of LPS in cells expressing TLR alone or TLR4 with MD-2 mutations. MD-2 is a crucial molecule for TLR4-mediated immune recognition (Ishihara et al., 2004), and thus *H. pylori* must avoid activating hTLR4-MD2 to establish long-term colonization of the gastric mucosa. However, TLR recognition of LPS remains controversial. While many studies suggest TLR4 as the primary TLR for LPS binding, others indicate that TLR2 is also a key TLR in LPS recognition (Francisco et al., 2021). TLR2 is capable of recognizing atypical forms of LPS and has been found to activate immune responses more strongly with hypo-antigenic HP-LPS than with highly antigenic LPS (Yokota et al., 2010; Nemati et al., 2017). TLR2 induces anti-inflammatory responses associated with IL-10 production and synergizes with TLR4 to enhance iNOS induction. This correlation may constitute a mechanism to stimulate the innate immune response (Uno et al., 2007). Furthermore, it has been confirmed in an *in vitro* study that TLR2 also acts as a key receptor involved in the process of IL-8 secretion by neutrophils triggered by HP-NAP (Wen et al., 2021). This multifaceted role of TLR2 could potentially open up new avenues for therapeutic interventions targeting immune modulation.

The lipopolysaccharide (LPS) O-antigen of *H. pylori* expresses Lewis (Le) antigens, typically including Le(x) or Le(y) epitopes (Kobayashi et al., 1993). These epitopes mimic human Lewis molecules and blood group antigens, which may facilitate adhesion and enable evasion of the host’s immune response, potentially leading to gastric atrophy (Moran, 2008; Wang et al., 2000). Studies have shown that inactivation of *H. pylori* Le antigens impairs colonization in murine models, while an increase in Le antigens has been observed in gastric cancer patients (Lee et al., 2006), underscoring the pivotal role of Le antigens in the progression of gastritis and gastric carcinoma. The dendritic cell-specific C-type lectin receptor DC-SIGN plays a critical role in directing immune responses (Engering et al., 2002; Appelmelk et al., 2003). Its interaction with *H. pylori* Lewis variants *in vitro* can diminish the production of interleukin-6 and the development of Th1 cells (Bergman et al., 2004). Surfactant protein D (SP-D), another C-type lectin, is upregulated in the gastric mucosa during infection and can specifically bind to *H. pylori*, reducing its activity by 50% (Murray et al., 2002). Moreover, *in vitro* culture of the escape mutant J178V, which evades SP-D binding through focused glycosylation of the LPS O-chain, has been documented (Khamri et al., 2005).

In addition to the low immunogenicity of *H. pylori* ‘s lipopolysaccharide (LPS), the evolutionary attenuation of the innate immune response by *H. pylori* flagellin proteins within the gastric environment may represent a further strategy for this chronic mucosal pathogen to evade and mitigate detrimental host responses. Despite the presence of TLR5, *H. pylori* flagellins do not appear to stimulate an innate immune response in gastric epithelial cells (Lee et al., 2003; Zou et al., 2021). Flagellin (Flg) monomers are recognized by TLR5, activating innate immunity via NF-κB and particularly inducing rapid upregulation of pro-inflammatory genes such as IL-6/8 (Zou et al., 2021; Hayashi et al., 2001; Gewirtz et al., 2001). Flg is a multidomain protein composed of D0, D1, and hypervariable (HV) regions. Although *H. pylori*’s Flg contains the D0 and D1 domains that stimulate TLR5 in other flagellins, it elicits no response from hTLR5. *In vitro* studies have shown that *H. pylori* flagellin (FlaA) is significantly less potent in activating TLR5-mediated interleukin (IL)-8 secretion than that from Salmonella Typhimurium—by a factor of 1000 (Gewirtz et al., 2004). The diverse post-translational glycosylation of *H. pylori* flagellin (Lee et al., 2003), along with manipulations of the N-terminal recognition domain of TLR5 (Gewirtz et al., 2004), may serve as mechanisms to alter the surface properties and receptor binding affinity of *H. pylori* flagellin. To elucidate the molecular mechanisms by which hFlg evades TLR5 detection, J.H. Kim et al. conducted mutational analyses and found that the lack of TLR5 activity in response to hFlg is primarily due to differences at R89T, L93K, and E114D. These variations disrupt the interaction of flagellin’s TLR5 activation hotspot residues with the cavity of TLR5’s LRR9 loop, thereby facilitating evasion of TLR5 detection (Kim et al., 2018).

Retinoic acid-inducible gene I (RIG-I)-like receptors (RLRs), a subfamily of pattern recognition receptors (PRRs), have been studied in the context of *H. pylori* infection. *H. pylori* actively suppresses STING and RIG-I signaling pathways, achieving immune suppression by downregulating the activation of IRF3, thus modulating the host’s innate immune response and promoting chronic gastric inflammation. In Sting-deficient mice, *H. pylori* infection leads to an enhanced Th17 inflammatory response and increased expression of the host immune regulator Trim30a (Dooyema et al., 2022). Furthermore, the IL-1β secretion process induced by *H. pylori* infection involves the collaboration of multiple immune system components, crucial for its immune evasion. The NLRP3 inflammasome, an intracellular protein complex capable of detecting pathogens, is activated following TLR-2 recognition of *H. pylori*. Subsequently, the inflammasome’s caspase-1 enzyme is activated to cleave pro-IL-1β, producing active IL-1β. The inflammasome facilitates the formation of an inflammatory environment by promoting pro-inflammatory factors, enhancing T cell activation and proliferation, and their differentiation into Tregs. This modulation of the immune response’s intensity and duration is a key pathway in *H. pylori*-induced inflammation (Tran et al., 2017).

### 3.2 Disrupting macrophage function

During *H. pylori* infection, dendritic cells are recruited to the gastric epithelium where, as primary antigen-presenting cells, they capture antigens and migrate to regional lymph nodes or the spleen. There, they present antigens to naive T cells, eliciting an immune response from the host. However, as part of the innate immune system, macrophages have limitations in eradicating *H. pylori*(Kronsteiner et al., 2016). Research has revealed that *H. pylori* has evolved multiple strategies to evade host immune surveillance, including inhibiting the phagocytic process, affecting immune cell polarization and metabolism, thereby promoting its persistent colonization and survival in the host’s stomach.

#### 3.2.1 Inhibiting the phagocytic function of macrophage

*H. pylori* disrupts the phagocytic process of macrophages, weakening their antigen-presenting capabilities and thereby sustaining infection. The bacterium’s lipopolysaccharide (LPS) binds to macrophage surface glycoproteins such as CD14 and TLR4, triggering an NF-κB-mediated inflammatory response and the release of cytokines including IL-1β, IL-6, and TNF-α. This process interferes with intracellular signaling pathways crucial to phagocytosis, such as protein tyrosine kinase, PI3K/Akt, and JAK/STAT pathways (Ciesielska et al., 2021), inhibiting the transmission of phagocytic signals and the process of endocytosis. The pathogenic factor CagA binds to macrophage surface receptors such as E-cadherin and β1 integrin, causing cytoskeletal reorganization through the activation of the SHP-2 phosphatase pathway, excessive phosphorylation of MAPK and PI3K, and thereby inhibiting the phagocytic pathway (Coulombe and Rivard, 2016). Studies also suggest that CagA might interfere with the fusion of phagosomes and lysosomes, and consequently obstruct the establishment of an acidic environment, by disrupting key components such as proton pumps and chloride channels, or affecting the assembly of acidification organs. This could alter the localization and assembly of related proteins, impacting the functionality of phagocytic cells (Osei-Owusu et al., 2021; Wu et al., 2005). Additionally, the binding of outer membrane proteins BabA and SabA to host cell surface receptors may affect the cytoskeleton and function, disrupting the creation of the acidic environment necessary for the fusion of phagosomes and lysosomes (Doohan et al., 2021).

Calcium ions, key regulators of cell signal transduction, are involved in lysosomal fusion and the formation of phagocytic vesicles (Pradhan et al., 2019). The binding of LPS, CagA, and VacA increases intracellular Ca^2+ concentration, disrupting the phagocytic and digestive processes within macrophages (Wong et al., 2017). Upon endocytosis, VacA disrupts intracellular calcium homeostasis, triggering the unfolded protein response (UPR) and endoplasmic reticulum (ER) dysfunction, which could even lead to mitochondrial dysfunction and cell death (Groenendyk et al., 2021; Akazawa et al., 2013). The complexities of these interactions and their impact on the host-pathogen dynamic warrant further investigation to elucidate.

#### 3.2.2 Promoting macrophage polarization

Macrophage phenotypes induced by *H. pylori* infection are diverse, playing roles in suppressing inflammation, modulating immune responses, and promoting angiogenesis as the infection progresses, thereby facilitating immune evasion and persistent chronic infection. In the early stages of infection, M1 macrophages characterized by high expression of NOS2, IL-1β, and TNF-α contribute to bacterial clearance. As the infection becomes chronic, the tissue-repairing M2 macrophages become more prevalent (Lu et al., 2020; Quiding-Järbrink et al., 2010). Yu-Kuan Huang et al. conducted an immunohistochemical analysis that revealed an increased presence of M2 macrophage phenotypes at the margins of gastric cancer, while noting a rise in M1 macrophages in the core regions (Huang et al., 2019). This differential distribution reflects the heterogeneity of inflammatory responses and the influence of specific tissue microenvironments. Hence, a thorough understanding of macrophage diversity and their dynamic changes during infection is imperative.

*H. pylori* induces the expression of heme oxygenase-1 (HO-1) in gastric epithelial monocytes and macrophages through the phosphorylation of CagA, which activates p38 MAPK and NRF-2 signaling pathways. Upregulated HO-1 activity enhances the expression of M2 polarization-associated genes, such as CD206, CD163, arginase-1, and chitinase-like protein 3, leading to a spindled anti-inflammatory phenotype change (Kang et al., 2021). This has been confirmed to defend against inflammation and enhance phagocytic function in a murine model of ulcerative colitis (Gwak et al., 2022). Therefore, HO-1 activity inhibits M1 markers and pro-inflammatory cytokines, aiding the transition from M1 to M2 and Mreg, attenuating immune responses, which may paradoxically benefit the survival of *H. pylori*. E. Alaluf reported that the absence of HO-1 enhanced the therapeutic effects of anti-tumor vaccines *in vivo* experiments by restoring CD8+ T cell proliferation and cytotoxicity (Alaluf et al., 2020), suggesting HO-1 as a potential therapeutic target. In the field of medicinal chemistry, metalloporphyrins were among the first molecules researched and utilized as non-selective inhibitors of heme oxygenase-1 (HO-1). Subsequent in-depth studies led to the development of imidazole-dioxolane derivatives, heralding the advent of the first generation of non-porphyrin-based, isozyme-selective inhibitors targeting HO-1 (Pittalà et al., 2013). Currently, several pharmaceuticals that indirectly modulate HO-1 activity have entered clinical trials or are undergoing them. For instance, tocilizumab, an IL-6 receptor antagonist, has been authorized for therapeutic use in multiple clinical trials (e.g., ChiCTR2000029765, NCT04317092, NCT04346355, NCT04335071) (Rossi et al., 2020). Although these agents do not directly target HO-1, their interaction with inflammatory pathways allows for an indirect regulation of HO-1 activity.

While the initial activation and inflammatory response of M1 macrophages during the early stages of *H. pylori* infection may aid in bacterial clearance, the continued secretion of inflammatory and chemotactic factors during persistent infection can exacerbate inflammation and promote bacterial colonization (Lu et al., 2020). *H. pylori*’s pathogenic factors, including urease and outer membrane lipopolysaccharides, stimulate the expression of iNOS, facilitating the conversion of L-arginine to nitric oxide (NO). This process leads to LPS+IFNγ-induced mitochondrial dysfunction and inhibition of the electron transport chain, intensifying the inflammatory response and impeding the transition from M1 to M2 macrophages (Cherdantseva et al., 2014). *In vivo* experiments have shown that inhibiting NO production can improve mitochondrial function and reprogramming towards the M2 macrophage phenotype (Van den Bossche et al., 2016), underscoring the role of NO as a critical regulator of M1 macrophage activity.

Moreover, heparanase is upregulated in *H. pylori* infection and promotes the polarization of macrophages towards the M1 phenotype by attracting pro-inflammatory and pro-tumorigenic cytokines such as IL-1, IL-6, TNF-α, MIP-2, and iNOS (Tang et al., 2021). Recent studies have evaluated the efficacy of three heparanase inhibitors—Roneparstat, PG545, and M402—in models of multiple myeloma, lymphoma, and melanoma. These investigations have demonstrated the potential therapeutic effects of these inhibitors in *in vivo* models. Specifically, M402, when used in combination with cisplatin or docetaxel, was able to inhibit spontaneous tumor metastasis and extend survival in the 4T1 mouse breast cancer model. These findings provide a crucial foundation for future clinical trials in *H. pylori* infection and gastric cancer models (Noseda and Barbieri, 2020; Weissmann et al., 2019; Zhou et al., 2011). As an endoglycosidase, heparanase cleaves heparan sulfate chains, thereby releasing growth factors and other bioactive molecules anchored to the extracellular matrix (ECM). This enzymatic action reduces the density and adhesive properties of the ECM, which in turn facilitates the migration of immune cells (Wu et al., 2015). This mechanism bears similarities to the functions of matrix metalloproteinases (MMPs), which are instrumental in the progression of gastritis and play a pivotal role in the invasiveness and metastasis of gastric cancer. During *H. pylori*-induced gastritis, pro-inflammatory cytokines such as TNF-α and IL-1β significantly stimulate the secretion of MMP-9 (Slomiany and Slomiany, 2016), MMP-10 (Costa et al., 2016), and MMP-13 (Sheibani et al., 2017). MMPs play a pivotal role in the development of gastritis by degrading proteins in the ECM, such as collagen and elastin. Increased expression of MMP impairs the structural integrity and function of the gastric mucosa, leading to the pathogenesis of gastritis (Jabłońska-Trypuć et al., 2016). However, macrophages in MMP7-deficient models exhibit high levels of IL-1β and iNOS mRNA, suggesting that MMP7 may attenuate *H. pylori*-induced gastric pathology and carcinogenesis by inhibiting M1 macrophage polarization (Krakowiak et al., 2015). Furthermore, in an *in vivo* study, MMPs were shown to recruit CD8+ T cells through the production of CXCL16 while simultaneously inhibiting the expression of Reg3a, E-cadherin, and ZO-1. This dual effect not only exacerbates the inflammatory response but also promotes tumor proliferation and invasion. Therefore, targeting MMP inhibition may represent a significant strategy in cancer therapy (Sheibani et al., 2017; Lv et al., 2019).

In the current pharmaceutical market, Periostat® (doxycycline hyclate) is the only MMP inhibitor approved by the FDA for the treatment of periodontal disease (Das et al., 2020). Despite challenges in early studies, clinical trials of MMP inhibitors for conditions such as gastric cancer, diabetic foot ulcers, and multiple sclerosis continue actively (Fields, 2019). Meanwhile, researchers have developed highly specific new MMP inhibitors, such as inhibitory monoclonal antibodies against MMPs. These antibodies, through their specificity in targeting MMPs, effectively inhibit the degradation of the extracellular matrix, a critical step in tumor metastasis. These inhibitors have shown potential anti-tumor activity in preclinical studies for breast cancer (Kwon, 2022).

#### 3.2.3 Modulating macrophage metabolism

Metabolic reprogramming of macrophages during *H. pylori* infection is a key mechanism for evading host immune surveillance. Macrophages adjust their immune function by modulating key metabolic pathways, including glycolysis, the tricarboxylic acid (TCA) cycle, fatty acid metabolism, and mitochondrial oxidative phosphorylation (Liu et al., 2021). Specifically, M1 macrophages inhibit oxidative phosphorylation and favor glycolysis to rapidly produce cytokines and reactive oxygen species to combat infection (Freemerman et al., 2014). Conversely, M2 macrophages enhance mitochondrial oxidative phosphorylation and utilize glucose to support anti-inflammatory actions, tissue repair, and clearance of post-infection debris (Huang et al., 2016). This metabolic dichotomy elucidates the distinct functions and strategies of M1 and M2 macrophages in the context of *H. pylori* infection, highlighting the central role of metabolic pathway reprogramming in regulating immune responses.

Arginase 2 (ARG2) is expressed in various cell types and participates in different metabolic processes by degrading L-arginine. In M1 macrophages, L-arginine is converted into nitric oxide (NO) and citrulline via iNOS, with NO production possessing antimicrobial properties that facilitate pathogen clearance. However, NO and its reactive nitrogen species may cause mitochondrial electron transport chain (ETC) deactivation, inhibiting the transition from M1 to M2 macrophages (Van den Bossche et al., 2016). *H. pylori* infection induces ARG2 expression, potentially leading to L-arginine depletion and reducing NO-dependent bactericidal activity (Lewis et al., 2010). *In vitro* experiments, ARG2-mediated L-arginine depletion diminishes CD3zeta expression, impacting T cell proliferation and cytokine production (Rodriguez et al., 2003; Zabaleta et al., 2004), thereby weakening Th1/Th17 T cell differentiation during chronic *H. pylori* infection and contributing to the pathogen’s immune evasion strategies. ARG2 deficiency results in a compensatory upregulation of *H. pylori*’s polyamine metabolism. Extensive research has confirmed the indispensable role of polyamines in immune responses to various pathogens and in regulating macrophage functions. They influence histone acetylation, thereby promoting gene expression associated with M1 macrophage activation and significantly increasing the production of the anti-inflammatory mediator NO (Brooks, 2013). Ornithine decarboxylase (ODC), the rate-limiting enzyme in polyamine metabolism, is also a key regulator of M1 macrophage activation and can be upregulated by *H. pylori*’s ERK and MYC signaling pathways (Cheng et al., 2005; Asim et al., 2010), with increased ODC in macrophages impairing the host’s response, favoring an environment with less inflammation conducive to persistent infection (Hardbower et al., 2017). Moreover, cystathionine gamma-lyase (CTH) influences polyamine metabolism upstream by regulating the accumulation of S-adenosylmethionine (SAM). Recent research using *in vivo* mouse models has found that knockout of the Cth gene in gastric macrophages (Gmacs) under *H. pylori* infection reduces the expression of genes and proteins related to cellular respiration, decreases DNA methylation, maintains redox balance, and enhances mitochondrial function. These changes suppress macrophage activity, promote M2 macrophage formation, and reduce the production of inflammatory mediators (Latour et al., 2022), potentially exacerbating chronic inflammation.

In a preclinical/Phase II study, INCB001158, as an arginase inhibitor, significantly increased L-arginine levels, reversing myeloid-derived suppressor cell-induced T cell immune suppression and offering a new avenue for advanced biliary cancer treatment (NCT03314935). Additionally, studies have pointed out that ornithine decarboxylase (ODC) is a potential target for esophageal squamous cell carcinoma treatment, with its inhibitor, difluoromethylornithine (DFMO), undergoing clinical trials for related cancers (He et al., 2017), and has been used in clinical trials for the treatment of pediatric neuroblastoma (Lozier et al., 2015). Concerning cystathionine gamma-lyase (CTH), a selective CTH inhibitor has been found to specifically target the active site, showing high sensitivity in IDH1 mutant astrocytomas (Echizen et al., 2023). These findings suggest that active research targeting these enzymes is underway and may become an important strategy for treating *H. pylori* infection or gastric cancer.

## 4 Evasion of adaptive immunity

The immune response elicited by *H. pylori* infection is characterized by a mixed reaction involving T helper cells, cytotoxic T cells, and NK cells (Larussa et al., 2015). In the early stages of infection, a population of CD8+ T cells with a tissue-resident memory (TRM) phenotype has been identified. These cells rapidly infiltrate the gastric mucosa and recognize specific antigens, including surface proteins and lipopolysaccharides. This mechanism provides a swift and effective immune response, which is crucial for controlling the infection. As the infection persists, the presence of TRM phenotype CD8+ T cells diminishes and is gradually replaced by CD4+ T cells, a shift that signifies a transformation in the immune response during the chronic phase of the infection (Koch et al., 2023).

### 4.1 Regulating Th1 cells

Following processing by antigen-presenting cells such as dendritic cells, *H. pylori* antigens are presented to CD4+ T cells, inducing the differentiation of naive CD4+ T cells into various subsets. Notably, Th1 cells are characterized by the production of interleukin-2 (IL-2) and interferon-γ (IFN-γ), assisting CD8+ T cells and promoting the generation of specific types of antibodies, thus eliciting a robust cellular response (Larussa et al., 2015; Chen et al., 2013). Conversely, during infection, the response of Th2 cells may be suppressed. An *in vivo* study administered different *H. pylori* preparations to BALB/c mice systemically at 14-day intervals. It was found that preparations containing LPS and recombinant CagA promoted the expression of IFN-γ, inducing a robust Th1-skewed immune response (Paydarnia et al., 2020). This might explain why *H. pylori* infection is often associated with a cellular immune response rather than a strong antibody response. However, *H. pylori* employs multiple mechanisms to interfere with this process, including diminishing antigen presentation, antigenic, variation, and secretion of immunosuppressive factors, thus reducing the efficacy of Th1 cells (Larussa et al., 2015).

*In vitro* experiments have shown that *H. pylori*’s virulence factor, VacA, interferes with immune responses by binding to the β2 integrin subunit (CD18) on T cells. VacA activates Cdc42 and Rac-1 through the phosphorylation of CD18 mediated by PKCη or PKCζ, inducing actin cytoskeletal rearrangement for endocytosis. The endocytosed VacA interferes with cellular signaling, alters cell morphology and function, and suppresses the immune response. Specific inhibition of PKCη or PKCζ phosphorylation can significantly reduce or completely block the uptake of VacA (Sewald et al., 2011). Further *vitro* research has shown that VacA enters activated T cells through its interaction with LFA-1, leading to vacuolation within the cells (Sewald et al., 2008). VacA can also induce G1/S cell cycle arrest and disrupt the nuclear translocation of NFAT, effectively preventing T cell proliferation (Gebert et al., 2003). cDNA microarray analysis has revealed that VacA specifically upregulates the mRNA expression of 60 genes in T cells and activates NF-κB in T cells via the classical pathway rather than the alternative pathway, indicating a selective and limited response of T cells to VacA (Takeshima et al., 2009). These findings provide mechanistic insights into the impact of VacA on host T cell functionality.

Moreover, *H. pylori* can directly restrict adaptive immune responses by limiting the nutrients essential for T lymphocytes. γ-glutamyl transpeptidase (GGT), a secreted protein, mediates the cleavage of extracellular glutathione and the production of reactive oxygen species (ROS) *in vitro* experiments. This activity consequently induces cell cycle arrest in lymphocytes (Schmees et al., 2007). It also disrupts T cell proliferation, activation, and effector cytokine expression by specifically depleting the extracellular space of glutamine through the inhibition of cMyc and IRF4 expression (Wüstner et al., 2015). Additionally, GGT possesses apoptotic-inducing activity (Shibayama et al., 2003), and interferes with Ras-dependent signaling, leading to G1 cell cycle arrest (Schmees et al., 2007), playing an indispensable role in bacterial colonization.

### 4.2 Regulating of Th17 cells

In recent research, Th17 cells have been substantiated as playing a pivotal role in the host’s defense against extracellular bacterial infections, notably *H. pylori*. The differentiation of Th17 cells is co-regulated by the cytokines IL-6, IL-23, and TGF-β. These factors drive the polarization of CD4+ T cells into Th17 cells, leading to the production of key cytokines such as IL-17A, IL-17F, and IL-22 (Larussa et al., 2015; Dixon et al., 2019). IL-17A, a signature cytokine of Th17 cells, also plays a crucial role early in the gastric mucosa of mice infected with *H. pylori* by recruiting and activating polymorphonuclear neutrophil (Kabir, 2011). A study by Numasaki et al. specifically illuminates the modulatory effects of IL-17 and IL-17F on Granulocyte-Macrophage Colony-Stimulating Factor (GM-CSF) production by lung microvascular endothelial cells when stimulated with IL-1β and/or TNF-α (Numasaki et al., 2004). This suggests that IL-17 can directly influence neutrophil behavior, favoring inflammatory responses. Upon activation, neutrophils not only recognize and engulf *H. pylori* through surface receptors such as TLR2 and TLR4 but also secrete leukocyte interleukins, including IL-12 and IL-2, and generate ROS for bactericidal effects (Alvarez-Arellano et al., 2007). This activation facilitates the polarization of Th1 responses and the maturation of dendritic cells. In an environment enriched with IL-12, the T-cell lineage is characterized by the robust production of IFN-γ and TNF-α, demonstrating significant cytotoxic activity (Tamassia et al., 2019; Fu and Lai, 2022). While studies have unveiled the capacity of neutrophils to capture and kill bacteria during infection, the intricacies of their role in infection and immune responses merit further exploration. A recent *in vivo* experiment revealed that histones, key components within the web-like DNA structures of Neutrophil Extracellular Traps (NETs), can directly activate T cells and promote the differentiation of Th17 cells. This process is mediated through TLR2 receptors on the T cell surface, leading to the phosphorylation of the crucial protein STAT3, a vital step in Th17 cell development (Wilson et al., 2022). This finding underscores the reinforcement of the Th17 response, shedding light on a critical aspect of immune system dynamics. Moreover, the role of IL-23 in gastric mucosal immunity has garnered attention, particularly its increased expression during *H. pylori* infection, suggesting that epithelial cells may contribute to shaping mucosal immune responses (Dewayani et al., 2021; Horvath et al., 2012). However, IL-23 does not directly stimulate the production of IFNγ; instead, it functions in memory cells, enhancing the Th17 cell response. Notably, IL-23 does not drive the differentiation of naïve CD4+ T cells towards Th17, but rather, regulates IL-17 secretion via the STAT3 pathway in the later stages of Th17 cell development (Dewayani et al., 2021; Caruso et al., 2007).

As early as 1998, *in vivo* studies had demonstrated that gastric epithelial cells (GECs) upregulate the expression of B7 costimulatory molecules (B7-1, B7-2, B7-H1, and B7-H2) during *H. pylori* infection. This upregulation helps diminish the activity of CD4+ effector T cells and increase Treg cells, thereby modulating the T cell response (Ye et al., 1997; Zhang et al., 2023). B7-H2 (ICOS ligand), a recent addition to the B7 receptor family, provides a costimulatory signal upon binding with ICOS, enhancing T cell activity. Current research have shown this in mice, CagA may downregulate B7-H2 expression through phosphorylation by p70 S6 kinase, aiding *H. pylori* in evading Th17-mediated immune responses and fostering chronic infection (Lina et al., 2013). This process contributes to the low responsiveness of CD4+ effector T cells and the accumulation of regulatory T cells. Additionally, cagA activates GECs, enhancing the binding of NF-κB to the REDD1 promoter via the MAPKp38 pathway, leading to upregulation of REDD1—Regulated in development and DNA damage responses-1—in *H. pylori*-infected gastric mucosa. Mouse model studies have further revealed that this action of cagA intensifies gastritis progression in non-bone marrow-derived cells. Furthermore, cagA increases the expression of CXCL1, promoting the migration of MHC II+ mononuclear cells and the secretion of IL-23, which collectively lead to Th17 cell polarization and IL-17A production, exacerbating inflammation induced by *H. pylori* (Yan et al., 2021). These findings deepen our understanding of cagA and its associated pathogenicity island (cagPAI) in the immune response elicited by *H. pylori*.

ILC-3 and Th17 cells exhibit a certain overlap in cytokine production, particularly in the generation of IL-17, which enables their synergistic role in regulating immune responses and inflammation. In *H. pylori* infection, Group 3 innate lymphoid cells play a pivotal role. Primarily located in the gut, ILC3s are crucial for maintaining intestinal immunity and the balance between the host and the microbial community (Dixon et al., 2016). These cells respond to microbial stimuli by producing IL-22 and/or IL-17, akin to Th17 cells. IL-22, predominantly produced by ILC3s, is essential for maintaining intestinal homeostasis. In interactions with *H. pylori*, ILC3s can limit bacterial proliferation by inducing the expression of antimicrobial peptides (AMPs). These AMPs, with potent antibacterial and anti-biofilm activities, cause bacterial cell membrane disruption, leading to cell lysis and death. Moreover, ILC3s contribute to the containment of commensal bacteria within lymphoid tissues and prevent systemic inflammation through the production of IL-22 (Elemam et al., 2021).

### 4.3 Regulating Treg cells

Regulatory T (Treg) cells, whose differentiation plays a modulatory role in immune responses, are predominantly influenced by Transforming Growth Factor-beta (TGF-β). TGF-β facilitates the conversion of CD4+ T cells into Treg cells, which are characterized by the expression of transcription factor FOXP3 and CD25, as well as the production of IL-10 (Utsch and Haas, 2016; Bagheri et al., 2016). In macrophages, VacA strongly induces the expression of cytokine genes, particularly the anti-inflammatory cytokines IL-10 and TGF-β. The TGF-β signaling pathway, in conjunction with microbial signals (such as short-chain fatty acids) and vitamin A, promotes the expression of Foxp3 in naive T cells, thereby influencing the immune response (Altobelli et al., 2019). Treg cells are essential in immune responses against *H. pylori* and in regulating inflammation to prevent host tissue damage, as they can suppress the cytokine production and proliferation of effector T cells. *In vivo* studies conducted by S. Raghavan et al. have demonstrated that athymic C57BL/6 nu/nu mice transfected with CD25(-) lymph node cells exhibited a significantly reduced colonization of *H. pylori* in the stomach, compared to mice transfected with CD25(+) LN cells. *In vitro* experiments further showed that splenocytes from mice receiving CD25(-) LN cells produced higher levels of interferon-gamma (IFN-γ) in response to *H. pylori* antigen stimulation and increased infiltration of CD4+ T cells and macrophages in the gastric mucosa (Raghavan et al., 2003), indicating a key role for Treg cells in modulating gastric mucosal inflammatory responses. Additionally, Treg cells isolated from *H. pylori*-infected patients, marked as CD4+/CD25^high, were shown to suppress the response of memory T cells. These findings suggest that in individuals infected with *H. pylori*, the CD4+ memory T cell response to the pathogen is diminished, and CD4+ CD25+ high Treg cells may facilitate chronic infection by suppressing this response (Lundgren et al., 2003), a mechanism of significant interest for vaccine development strategies.

*In vivo* experiments, neonatal mice infected with *H. pylori* demonstrated that two virulence factors of the bacterium, VacA and γ-glutamyl transpeptidase (GGT), can reprogram dendritic cells (DCs). This reprogramming involves inhibiting the maturation of DCs and promoting the development of Treg characteristics, leading to immune tolerance. Post-infection, DCs exhibited a propensity to induce Treg differentiation rather than eliciting Th1 or Th17 responses, without producing pro-inflammatory factors (Oertli et al., 2012; Wang et al., 2010). This tolerogenic behavior of DCs was ubiquitous across different mouse strains and occurred independently of α/β T cells (Oertli et al., 2013). However, *H. pylori* mutants lacking GGT or VacA were unable to inhibit lipopolysaccharide (LPS)-induced DC maturation or induce Treg properties in immature T cells, suggesting that these two virulence factors might independently drive DC tolerance (Oertli et al., 2013). Furthermore, the virulence factors VacA and CagA can promote Treg cell infiltration by activating the wnt/β-catenin pathway and upregulating CCL28, which is associated with β-catenin expression in gastric adenocarcinoma. In an MNU-induced gastric cancer mouse model, antibodies targeting CCL28 significantly reduced Treg cell infiltration and tumor growth (Ji et al., 2020; Korbecki et al., 2020), representing a novel and promising therapeutic approach for the future.

Within the host, there is often a delicate balance maintained between Th17 and Treg cells, which aids in sustaining an appropriate immune response. *H. pylori* skews this Th17/Treg balance towards Tregs, attenuating the Th17 response and thus impeding the clearance of this pathogen (Kao et al., 2010). The inactivation of transcription factor c-MAF in the Treg cell region not only affects the differentiation and function of bacterium-specific iTreg cells but also leads to the accumulation of inflammatory TH17 cells specific to H. hepaticus (Xu et al., 2018). Depletion of Tregs enhances the *H. pylori*-specific Th17 response, which correlates with a reduction in bacterial density.

## 5 *H. pylori* modulates miRNA to regulate host immunity

Alterations in epigenetic mechanisms, such as aberrations in DNA methylation and various histone modifications including acetylation, methylation, and phosphorylation, are pivotal in the pathogenesis of human cancers. They exert an impact on gene expression through the silencing of various tissue-specific genes and the methylation of imprinted genes (Pajares et al., 2021; Audia and Campbell, 2016). Additionally, the expression of microRNAs (miRNAs) is also regulated by epigenetic variations. Changes in miRNA expression in gastric epithelial cells induced by *H. pylori* infection are closely linked to the progression of gastric mucosal lesions and play a dual role in cancer development as tumor suppressors or oncogenes, underscoring their importance in gene regulation (Săsăran et al., 2021).

MiRNAs have been described in multiple studies as modulators of TLR signaling, influencing the release of pro-inflammatory cytokines and signaling cascade proteins by binding to the 3’-UTR regions of TLRs or acting as transcriptional regulators. During the process of *H. pylori* infection, particularly under the influence of CagA-positive strains, the expression of the let-7 family of miRNAs in gastric epithelial cells is inhibited (Hayashi et al., 2013). This downregulation directly leads to the suppression of TLR4 and NF-κB signaling pathways, consequently reducing the inflammatory response (Teng et al., 2013). This highlights the role of miRNAs as direct ligands affecting the activity of TLR signaling pathways. Concurrently, the expression of miRNA-146 and miRNA-155 is upregulated in gastric epithelial cells, macrophages, and T cells (Xie et al., 2014; O'Connell et al., 2007). This upregulation inhibits the NF-κB signaling pathway and the release of the pro-inflammatory cytokine IL-8, while also modulating T cell activity and the differentiation of Th1 and Th17 cells (Möhnle et al., 2015; Oertli et al., 2011). This dual regulatory function aids in balancing immune responses, facilitating persistent *H. pylori* infection and resistance to apoptosis. Therefore, the crosstalk between TLRs and miRNAs is crucial for ensuring an appropriate immune response to pathogens. *H. pylori* infection also triggers an upregulation of specific miRNAs in macrophages, notably let-7i-5p, miR-146b-5p, and miR-185-5p. These miRNAs, by suppressing CIITA, lead to a decreased expression of HLA class II molecules. This reduction impairs the macrophages’ capacity for bacterial processing and antigen presentation to Th cells, consequently attenuating the immune response against *H. pylori* (Codolo et al., 2019). Moreover, *H. pylori* infection downregulates miR-4270, promoting the expression of CD300E, which further affects MHC-II functionality and reduces immune presentation (Pagliari et al., 2017). These miRNAs, due to their role in regulating innate immunity and inflammatory responses during *H. pylori* infection, hold promise as biomarkers for the disease.

Regarding miRNA-155, an LNA-modified antisense inhibitor, MRG-106, is currently in phase II clinical trials for patients with cutaneous T-cell lymphoma and mycosis fungoides. This miRNA has been recognized for its role in inflammation, and its inhibition could enhance the differentiation of brown adipocytes (Kornmueller et al., 2022). There is also evidence that miRNA mimics of miR-146 may be promising for anti-inflammatory therapy in further preclinical studies (Comer et al., 2014), while Mesenchymal Stem Cells (MSC) -derived exosomes transduced with miR-146a/miR-155 have shown potential immunomodulatory effects in experimental models (Tavasolian et al., 2020). Although no specific therapeutic applications in clinical trials have been found for let-7i-5p, miR-146b-5p, and miR-185-5p, these miRNAs have been identified as targets for mitigating the adverse effects of macrophage antigen presentation (Deng et al., 2022), suggesting they may play a role in modulating inflammatory responses. Furthermore, let-7i-5p is considered to be involved in various signaling pathways related to cell morphology and migration, and some miRNA-based drugs, such as anti-Let-7f, have been tested in experimental models of central nervous system injury (Tavasolian et al., 2020).

## 6 *H. pylori* vaccine

Antibiotics serve as the primary modality for treating *H. pylori* infections. Unfortunately, this bacterium has demonstrated resistance to multiple antibiotics, a phenomenon largely attributable to genetic mutations. These mutations not only confer resistance but may also enhance the virulence of the bacteria (Lin et al., 2023). For instance, mutations in the 23S rRNA gene of *H. pylori* can impede the binding of clarithromycin to the ribosomal subunit, leading to resistance to this drug (Albasha et al., 2021). Generally, antibiotics inhibit bacterial growth by interfering with DNA gyrase or topoisomerase activities. In *H. pylori*, mutations in the gyrA and gyrB genes could alter the structure or function of DNA gyrase and topoisomerase, resulting in resistance to antibiotics such as levofloxacin (Fauzia et al., 2023). Since the 1990s, studies have confirmed that oral vaccines can provide protection before and after *H. pylori* infection (Czinn et al., 1993), and the development of effective vaccines is viewed as a promising strategy to prevent gastric cancer. Vaccine antigen research has focused on *H. pylori* virulence factors, such as urease, cytotoxin-associated gene A (CagA), vacuolating cytotoxin A (VacA), neutrophil-activating protein (NAP), and heat shock proteins (HSPs) (Tshibangu-Kabamba and Yamaoka, 2021; Corthésy-Theulaz et al., 1995). Administration via various routes including sublingual, intranasal, and gastric mucosal delivery simultaneously stimulates both mucosal and systemic immune responses (Sjökvist Ottsjö et al., 2017). However, the capability of these vaccines to directly neutralize all virulence factors of *H. pylori*, including CagA and VacA proteins, remains an ongoing research topic. The efficacy of *H. pylori* vaccines largely depends on their ability to elicit effective immune responses to combat the bacteria. T.H. Ermak et al. demonstrated in murine models that the protective effect induced by urease vaccines primarily relies on MHC class II-restricted cell-mediated immune mechanisms. Furthermore, even in B-cell knockout mice, the vaccine provided protection comparable to that in normal mice, underscoring the significance of cell-mediated immunity in the immunogenic response induced by urease vaccines (Ermak et al., 1998).

Inducing potent cellular immunity, particularly Th1 and Th17 responses, is crucial for the eradication of *H. pylori*. Kao and others demonstrated that vaccination against *H. pylori* could successfully stimulate a Th17 immune response in mice lacking antibodies or Th2 responses, inversely correlated with *H. pylori* colonization (Kao et al., 2010). The vaccine-induced Th17 cell response, associated with the gastric aggregation of neutrophils, plays a central role in the clearance of *H. pylori* (DeLyria et al., 2009). Another study pointed out that the immune protection induced by specific Th epitopes in BALB/c mice relies on the production of IFN-γ by CD4+ T cells (Li et al., 2015), which appears to depend on the antigen’s characteristics and its interaction with the immune system. Additionally, dendritic cells play a pivotal role in vaccine-induced immune protection through the expression of protease-activated receptor 2 (PAR2) (Velin et al., 2011), which is crucial for balancing tissue damage and immune response.

In addition to traditional mucosal adjuvants such as Cholera Toxin and Escherichia coli heat-labile enterotoxin (Bowman and Clements, 2001), to address the issues of enzymatic degradation and extreme pH encountered in oral *H. pylori* vaccines, researchers have explored a variety of vaccine designs, including optimization of antigen and adjuvant technologies. For instance, the CWAE multivalent subunit vaccine, containing selected B and Th cell epitopes and the UreB subunit, can induce a mixed CD4+ T cell response and high antibody levels (Guo et al., 2017). D,L-lactide-co-glycolic acid (PLGA) and nanoparticles (NPs) as vaccine carriers enhance the stability and immunogenicity of vaccines (Waeckerle-Men and Groettrup, 2005), offering significant advantages in improving stability, extending circulation time, and enhancing safety and efficacy (Mitchell et al., 2021). In one study, an acid-resistant HP55/PLGA nanoparticle oral vaccine system elicited high levels of specific antibodies and memory T cell responses in mice, significantly reducing bacterial load and achieving 43% complete protection (Tan et al., 2017), confirming the significant potential of encapsulating vaccine antigens to enhance oral vaccine effectiveness.

Although these experimental outcomes have demonstrated positive responses *in vitro*, clinical trials have largely yielded disappointing results. A Phase I/II clinical trial (NCT00736476) assessed the efficacy of a vaccine composed of vacuolating cytotoxin A (VacA), cytotoxin-associated antigen (CagA), and neutrophil-activating protein (NAP) for preventing infection with cagA-positive strains post-intramuscular immunization. Findings at 12 weeks post-infection revealed a 68% clearance rate in the vaccinated group compared to a 60% rate in the placebo group (Malfertheiner et al., 2018), which does not conclusively prove the vaccine’s effectiveness due to natural immune responses elicited by *H. pylori.* In another Phase III trial (NCT02302170), a prophylactic vaccine containing urease and Escherichia coli heat-labile enterotoxin B fusion protein was shown to reduce *H. pylori* infection rates in children aged 6-15 years. The vaccine’s efficacy was 71.8% (95% CI 48.2-85.6) within the first year. However, participants had to fast for at least two hours and ingest sodium bicarbonate two minutes before vaccination. The study also suggested that the vaccine’s long-term efficacy might diminish, possibly necessitating regular revaccination (Zeng et al., 2015). Most candidate vaccines, despite demonstrating promising immunoprotective effects in animal studies, have shown limited efficacy in human clinical trials. This discrepancy is largely attributed to the high genetic diversity of *H. pylori*, especially in terms of antigenic variability of its virulence factors (Kabamba et al., 2018). This variability leads to low conservation of some antigens across different strains, enabling these antigens to more easily evade recognition by the host immune system. Consequently, they can only provide partial protection and exhibit poor immunogenicity. This characteristic poses significant challenges in the development of effective vaccines against *H. pylori*.

## 7 Conclusion

*H. pylori* is a significant gastric pathogen directly linked to various gastric diseases, such as peptic ulcers and gastric cancer. In China, the prevalence of *H. pylori* infection is notably high, posing a significant health challenge to the general population. Consequently, elucidating *H. pylori* ‘s pathogenic mechanisms and finding effective treatments have become focal points in medical research. Notably, how *H. pylori* evades the host immune system has emerged as a research hotspot in recent years. Despite an immune response capable of clearing most pathogens, *H. pylori* has evolved a suite of mechanisms to circumvent both innate and adaptive immune responses, enabling the bacterium to persistently infect and colonize the host. Numerous studies have revealed *H. pylori* ‘s critical role in escaping the host immune system, particularly in ex *vivo* preclinical models. *H. pylori* virulence factors can bind to host macrophage surface receptors, alter macrophage polarization states, disrupt phagocytic function, and induce differentiation of T cells into immunosuppressive regulatory T (Treg) cells. In this process, multiple potential therapeutic targets against *H. pylori* have been identified. Moreover, the development of vaccines targeting *H. pylori* infection has demonstrated potential application prospects. In summary, a thorough understanding of the interactions between *H. pylori* and the host immune system is crucial for developing targeted treatments and vaccines. Future research will focus on elucidating the intricate mechanisms of interaction between *H. pylori* and the host immune system, as well as conducting extensive experiments to evaluate the clinical efficacy of targeted therapeutics and *H. pylori* vaccines.

## Author contributions

JF: Conceptualization, Data curation, Writing – original draft. JZ: Supervision, Validation, Visualization, Writing – review & editing. HX: Supervision, Validation, Visualization, Writing – review & editing.
